# Two new species of the genus *Morphostenophanes* Pic, 1925 from Yunnan, China (Coleoptera, Tenebrionidae, Stenochiinae, Cnodalonini)

**DOI:** 10.3897/zookeys.1282.176631

**Published:** 2026-06-15

**Authors:** Hao He, Fei-Yan Meng, Shi-Jian Yang

**Affiliations:** 1 School of Life Science, Yunnan Normal University, Kunming 650500, China School of Life Science, Yunnan Normal University Kunming China

**Keywords:** Darkling beetles, distribution, intraspecific variant, species groups, taxo­nomy

## Abstract

Two new species of darkling beetles of the genus *Morphostenophanes* Pic, 1925 (Coleoptera, Tenebrionidae, Stenochiinae, Cnodalonini) are described from Yunnan, China: *M. jiaoziensis***sp. nov**. and *M. yangshijiani***sp. nov**. Additionally, the female of *M. bannaensis* Zhou, 2020 is described for the first time. A new species group, the *yangshijiani* group, is proposed. Distributional maps for the two new species and an updated key to the *elegantulus* and *yangshijiani* groups are provided. Moreover, intraspecific variant individuals of the two new species are described, and the geographical distribution, elevational ranges, and phenological differences of these species are discussed. The factors contributing to the differences in populations of *M. jiaoziensis* are analysed.

## Introduction

The genus *Morphostenophanes* Pic, 1925 is an arboreal group of darkling beetles (Coleoptera, Tenebrionidae) endemic to the Oriental Region. Its species are medium-sized to large beetles with an elongate body and metallic lustre. The genus is assigned to the tribe Cnodalonini of the subfamily Stenochiinae (Masumoto and Bečvář 2008; Bouchard and Bousquet 2021).

Pic (1925) established *Morphostenophanes* based on *M. aenescens* Pic, 1925, the type species, which was collected from Yunnan, China. Kaszab (1941) described a second species, *M. papillatus* Kaszab, 1941, from Chongqing. Kaszab (1960) established the genus *Promorphostenophanes* based on a single female specimen from Yunnan and designated *P. atavus* Kaszab, 1960 as its type species, and later he (Kaszab 1980) described two subspecies of *P. atavus* from Vietnam and Myanmar: *P. atavus vietnamicus* Kaszab, 1980 and *P. atavus birmanicus* Kaszab, 1980. Masumoto (1990) described *P. koyamai* Masumoto, 1990 from Chiang Mai, Thailand and later (Masumoto 1998) described *M. tanikadoi* Masumoto, 1998 and two subspecies of *M. jendeki* Masumoto, 1998 (*M. j. jendeki* and *M. j. similis* Masumoto, 1998) from Sichuan and Yunnan. Masumoto and Bečvář (2008) synonymized *Promorphostenophanes* with *Morphostenophanes*, elevated the two subspecies of *M. atavus* to species rank as *M. birmanicus* (Kaszab, 1980) and *M. vietnamicus* (Kaszab, 1980), and treated *M. koyamai* (Masumoto, 1990) as a junior synonym of *M. birmanicus* (Kaszab, 1980). Gao and Ren (2009) described *M. cuproviridis* from Guizhou and *M. tuberculatus* from Yunnan. Zhou (2020) redefined and comprehensively revised the genus, describing 17 new species and three new subspecies, and systematically classified the species of the genus into six species groups. Huang (2025) described *M. orichalcos* Huang, 2025 from northern Vietnam, bringing the total number of species-group taxa in the genus to 32.

The genus *Morphostenophanes* is distributed in China, Thailand, Vietnam, and Myanmar, but China’s Yunnan province is the core area of its distribution. This study describes two new species from Yunnan, bringing the total number of *Morphostenophanes* species recorded in this province to 27. We also provide an updated key, distribution maps, and habitat photographs for the two new species.

## Materials and methods

### Preparation of figures

Habitus photographs were taken using a Sony α7RIII digital camera with Laowa 60 mm f/2.8 MF Macro 2:1 lens; photographs of smaller characters were taken using the same camera equipped with a Laowa 25 mm f/2.8 Ultra Macro 2.5–5.0× lens and stacked using Helicon Focus v. 8.1.0. The base map for the distribution map (Fig. 4A) was downloaded from SimpleMappr (https://www.simplemappr.net), and the base map for the elevation map (Fig. 4B) was obtained from Geospatial Data Cloud (http://www.gscloud.cn/). Both base maps were modified using Global Mapper v. 24.1. The photographs in Figs 9, 10, 11 were taken using an iPhone XR smartphone; all photographs were edited in Adobe Photoshop 2023 (v. 24.0.0).

Abbreviations for collections in this study are: **CHH**—Collection of Hao He, Qujing, Yunnan, China; **KIZ**—Kunming Institute of Zoology, Chinese Academy of Sciences, Kunming, China.

### Data treatment

The following abbreviations are applied in measurements (Zhou 2020): **EL**—length of elytra along midline; **EW**—maximum width of elytra; **OI**—ocular index; **PL**—length of pronotum along midline; **PW**—maximum width of pronotum.

Body length was measured from anterior margin of clypeus to elytral apex; body width equals EW. All measurements are in millimetres (mm).

## Results

### Taxonomy

***Morphostenophanes* Pic, 1925**

***Morphostenophanes elegantulus* species group (*elegantulus* group) Zhou, 2020**

#### Morphostenophanes jiaoziensis sp. nov.

Taxon classificationAnimaliaColeopteraTenebrionidae

C8064654-8A67-5B8C-9575-DFB95E461713

https://zoobank.org/E60BC793-86FC-478F-9A76-7D5375655DC7

**Figure 1.**
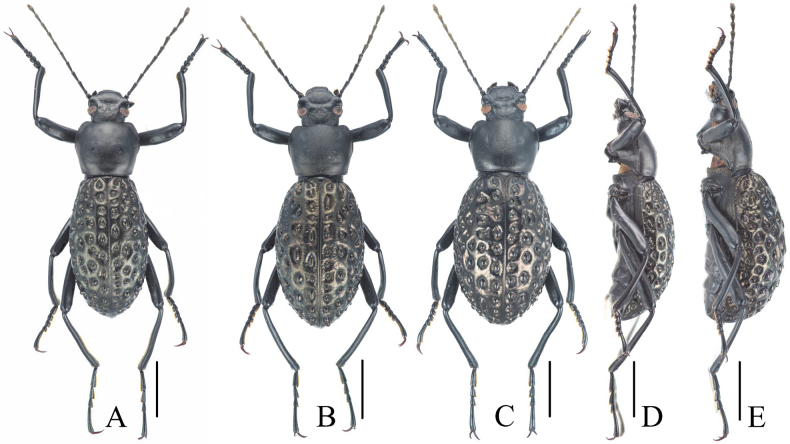
Habitus of *Morphostenophanes jiaoziensis* sp. nov. **A**. Male, dorsal view; **B, C**. Female, dorsal view; **C**. Variant; **D**. Male, lateral view; **E**. Female, lateral view. Scale bars: 5 mm (**A–E**).

**Figure 2.**
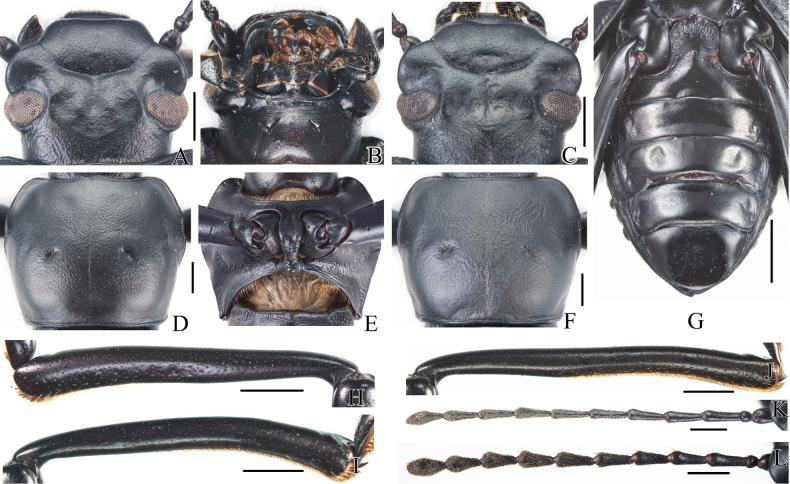
External characters of male *Morphostenophanes jiaoziensis* sp. nov. Heads: **A**. Dorsal view; **B**. Ventral view; **C**. Dorsal view. Prothoraces: **D**. Dorsal view; **E**. Ventral view; **F**. Dorsal view; **G**. Abdomen. Tibiae: **H**. Protibia, dorsal view; **I**. Mesotibia, dorsal view; **J**. Metatibia, dorsal view. Antennae: **K**. Dorsal view; **L**. Dorsal view. **C, F, L**. Paratype (northern population). Scale bars: 1 mm (**A–F, H–L**); 2 mm (**G**).

**Figure 3.**
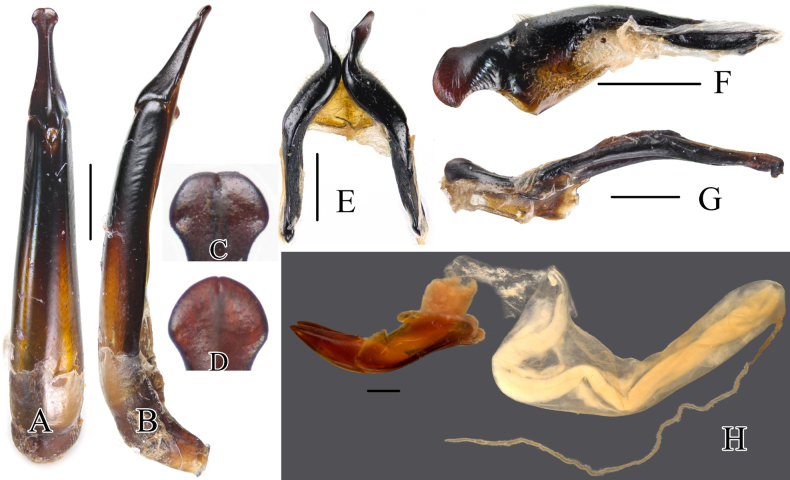
Anatomical structures of *Morphostenophanes jiaoziensis* sp. nov. Aedeagus: **A**. Dorsal view; **B**. Lateral view; Enlargements of paramere apices: **C**. Holotype; **D**. Paratype from northern population. Male sternite VIII: **E**. Dorsal view; **F**. Lateral view; **G**. Spiculum gastrale, lateral view; **H**. Female reproductive organs. Scale bars: 1 mm (**A–H**); **C**, **D** not to scale.

Figs 1, 2, 3, 9B, 10C, 11D

##### Type materials.

**Figure 4.**
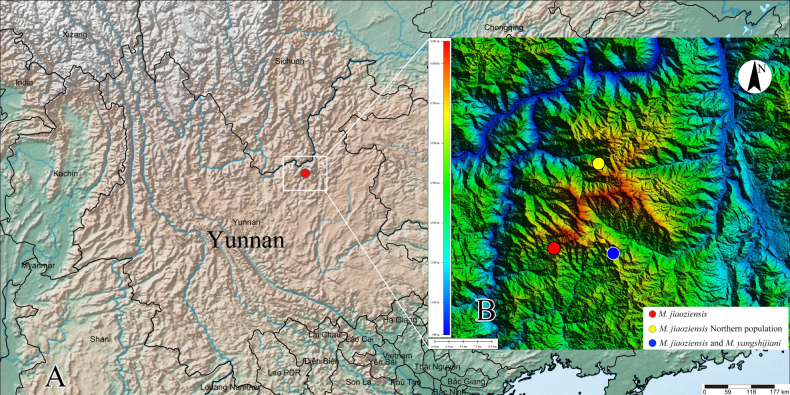
**A**. Distribution of *Morphostenophanes jiaoziensis* sp. nov. and *M. yangshijiani* sp. nov. **B**. Close-up of distribution of the two species.

**China**: Yunnan: • ♂ (**holotype**, KIZ), Kunming City, Luquan County, Jiaozishan National Nature Reserve, 26°04'04.39"N, 102°49'49.52"E (Fig. 4B, red dot), alt. 3100 m, 12.vi.2022, Hao He leg.; **paratypes** • 6♂, 3♀ (CHH): 1♂, 26°12'02.33"N, 102°54'01.48"E (Fig. 4B, yellow dot), alt. 3300 m, 22.iv.2022, Hao He leg.; • 1♀, same data as holotype; 26°03'34.56"N, 102°55'24.28"E (Fig. 4B, blue dot), alt. 2900 m: 2♀, 15.vi.2023, Jun-dong Xu leg.; • 1♂, 23.vi.2024, Hao He leg.; • 4♂, 20.v.2025, Hao He leg.

##### Diagnosis.

Body medium-sized to large, black with silvery-grey metallic reflection. Frontal midline depressed, extending to clypeus. Pronotum inversely trapezoidal, strongly constricted between pronotum and elytra. Elytra with rows of striae formed by round and oval segments; encircled areas strongly convex. Apex of parameres bent ventrad in lateral view.

##### Description.

**Male** (Fig. 1A, C). Body medium-sized to large, black, antennae and claws reddish brown. Body elongate, length 19.3–21.3 mm, width 7.3–7.5 mm, strongly convex.

***Head*** (Fig. 2A, E) transversely subquadrate, black, densely and finely punctate. Clypeus transversely heptagonal (width/length = 1.82); disc weakly convex, anteriorly slightly curved downward; anterior margin nearly straight, slightly convex in middle; frontoclypeal sulcus well marked, more deeply concave at middle, fading laterally, broadly U-shaped. Genae strongly raised, densely and finely punctate, strongly and roundly produced anterolaterally; outer margin deeply notched between gena and clypeus. Eyes transversely reniform, strongly convex laterally; OI = 50.1–56.4; inner ocular sulci deeply grooved along inner margins, becoming shallower and broader posteriorly. Frons broad, declivous anteriorly towards clypeus, with a distinct median sulcus that extends to frontoclypeal sulcus.

***Mentum*** (Fig. 2B) inversely trapezoidal; lateral margins slightly rounded, rising gradually from sides to middle; medial surface sparsely and coarsely punctate, with several large pores bearing long setae.

***Antennae*** (Fig. 2K) slender, deep brownish black, extending to basal fifth of elytra, with antennomeres weakly thickened to apices; relative lengths of antennomeres: 0.60: 0.36: 1.03: 0.96: 1.00: 1.00: 1.01: 0.93: 0.89: 0.87: 0.99.

***Pronotum*** (Fig. 2D) black, inversely trapezoidal; PW/PL = 1.18–1.30. Posterior half gradually narrowed to base, densely and finely punctate, widest near middle; anterior angles broadly rounded, posterior angles obtuse; anterior marginal border distinct, medially slightly convex and bisinuate; anterior half of lateral margin rounded, weakly sinuous; lateral margins sharply defined and thickened; posterior half not visible in dorsal view; posterior margin marked and nearly straight, thinning medially, and weakly concave at middle; disc strongly convex medially, with a pair of distinct shallow depressions medially, presenting a thin, longitudinal impression along midline. Scutellum widely triangular, finely and sparsely punctate.

***Elytra*** elongate-oval, black with silvery-grey metallic reflection, widest medially, EL/EW = 1.78–1.89, strongly convex, highest near middle, with rows of striae formed by round and oval segments; encircled areas strongly convex, intervals strongly convex, intervals finely granulate and densely covered with fine wrinkles, sparsely and finely punctate.

***Prosternum*** (Fig. 2E) shagreened, finely and sparsely punctate; prosternal process thickened, with coarse wrinkles extending ventrad; apex truncate; hypomeron rugulose. Metasternum glossy, finely punctate.

***Abdomen*** (Fig. 2G) depressed, with surface smooth and finely punctate. Sternites III–VI wrinkled on lateral margins; sternites III and IV with or without a vague posteromedian depression; sternite V with lateral median impressions.

***Legs*** slender, black. Protibiae (Fig. 2H) nearly straight, gradually thickened toward apex; apical third faintly curved; apical half of inner margins weakly pubescent. Mesotibiae (Fig. 2I) strongly curved in apical third, apical 3/5 of inner margins weakly pubescent, gradually thickened toward apex. Metatibiae (Fig. 2J) nearly straight; apical third faintly curved and bisinuate; apical 2/3 of inner margins densely pubescent.

***Aedeagus*** (Fig. 3A, B) elongate, curved in lateral view; parameres slender, about 0.20 as long as total length, ridged along dorsal midline, apex dilated, bluntly hastate, both sides slightly recurved dorsally. Laterally, superior margin of sternite VIII (Fig. 3E, F) weakly reflexed; apical lobes of sternite VIII with rounded exterior and inferior margins.

**Female** (Fig. 1B, C, E) stouter than male, length 20.5–22.5 mm, width 7.5–9.0 mm, OI = 55.0–55.6. ***Pronotum*** inversely trapezoidal, with a pair of obscure median impressions, PW/PL = 1.31–1.38. ***Elytra*** ovate and more convex than in male, EL/EW = 1.56–1.80. ***Abdomen*** straight in lateral view; sternites V with a pair of elongate depressions anterolaterally; sternite VII with a pair of vague median impressions. ***Ovipositor*** elongated, gradually narrowing posteriorly, with acute apex.

##### Comparative notes.

*Morphostenophanes jiaoziensis* most closely resembles *M. furvus* Zhou, 2020. Both species are black and the elytra bear rows of striae formed by round or oval segments., but the new species can be distinguished from *M. furvus* by having a median frontal sulcus (absent in the latter), more convex elytra with a stronger metallic lustre (weaker in the latter), an inversely trapezoidal pronotum with a rougher disc (smooth in the latter), a bisinuate anterior margin (straight in the latter), and a pair of lateral depressions (absent in the latter).

##### Variability.

One female paratype (Fig. 1C) has a more robust habitus, as well as broader and more rounded elytra (EL/EW = 1.56). Compared to males from the northern population in the Jiaozi Mountains, males from the southern population have finer, sparser punctures on the head (Fig. 2C) and a shorter clypeus (length/width = 2.09) with a distinct clypeal transverse impression and an outer margin that is slightly notched between the gena and the clypeus. The antennae (Fig. 2L) are shorter, thicker, and darker in colour; the antennomeres strongly thickened apically, and the relative lengths of antennomeres are as follows: 0.51: 0.30: 0.87: 0.85: 0.83: 0.87: 0.89: 0.87: 0.84: 0.83: 0.91; the antennomeres reach the basal fifth of the elytra; pronotal (Fig. 2F) punctation is finer and sparser, the anterior marginal border is straight, and the lateral marginal border is rounded; the hypomeron has more numerous rugae. In the holotype malem the apex of the aedeagal paramere slightly truncate (Fig. 3C).

##### Comments.

All specimens of this species were collected from the areas surrounding the Jiaozi Mountains. One sampling site was on the northern side of the mountains, and two sites were on the southern side (Fig. 4B). These northern and southern populations are separated by the Jiaozi Mountains, which exceed 4,000 m in elevation; the surrounding river valleys are below 1000 m. This geographical barrier is likely a significant factor contributing to the morphological differences observed between the two populations. However, specimens from the two southern collection sites show very little inter-individual variation.

This species possesses certain characteristics of the *aenescens* group, such as the presence of a frontal median sulcus on the frons, and a posteromedian depression on ventrites III and IV. However, we assign this species to the *elegantulus* group based on its elytra, which is broadest at the middle, the slender female ovipositor, and the dense pubescence on the metatibia; we suggest that there is a close phylogenetic affinity between the *aenescens* and *elegantulus* groups.

##### Distribution.

(Fig. 4A). China: Yunnan, Jiaozishan National Nature Reserve.

##### Etymology.

The new species is named after its type locality, the Jiaozi Mountains.

###### *Morphostenophanes yangshijiani* species group (*yangshijiani* group)

The *M. yangshijiani* species group is characterized by the following combination of characters: body small; elytra bear rows of striae formed by round and oval segments; male protibia curved; parameres apically expanded and obtusely spearhead-shaped; ovipositor shortened, abruptly narrowing from apical third.

#### Morphostenophanes yangshijiani sp. nov.

Taxon classificationAnimaliaColeopteraTenebrionidae

B1A0D8B7-53D0-598E-AF02-9CC76A55ACFA

https://zoobank.org/98658ACE-F90D-43BB-8BBD-576355769ADA

Figs 5, 6, 7, 11C

##### Type materials.

**China**: Yunnan: • ♂(**holotype**, KIZ), Kunming City, Luquan County, Jiaozishan National Nature Reserve, 26°03'34.56"N, 102°55'24.28"E (Fig. 4B, blue dot), alt. 2900 m, 15.vi.2023, Hao He leg.; **paratypes** • 1♂, 6♀ (CHH), same collection data as holotype: 3♀, 15.vi.2023, Hao He leg.; • 2♀, 30.v.2024, Hao He, Jing-Hong Dai leg.; • 1♂, 1♀, alt. 2900 m, 20.v.2025, Hao He leg.

##### Diagnosis.

Small; antennae, tibiae, and tarsi green in dorsal view, claws reddish brown. Elytra with rows of striae formed by round and oval segments, encircled areas strongly convex.

##### Description.

**Figure 5.**
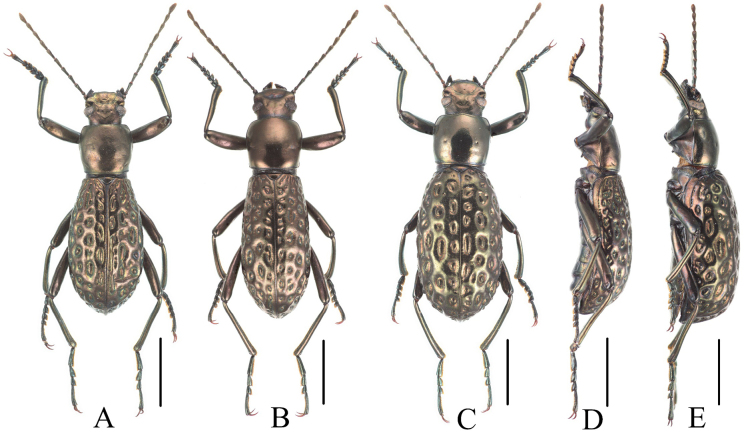
Habitus of *Morphostenophanes yangshijiani* sp. nov. **A**. Male, dorsal view; **B**. Male, dorsal view (paratype, variant); **C**. Female, dorsal view; **D**. Male, lateral view; **E**. Female, lateral view. Scale bars: 5 mm (**A–E**).

**Male** (Fig. 5A, B, D) copper-coloured, with a strong green metallic lustre; body elongate, length: 16.3–17.8 mm, width: 5.2–5.4 mm, noticeably constricted between pronotum and elytra.

**Figure 6.**
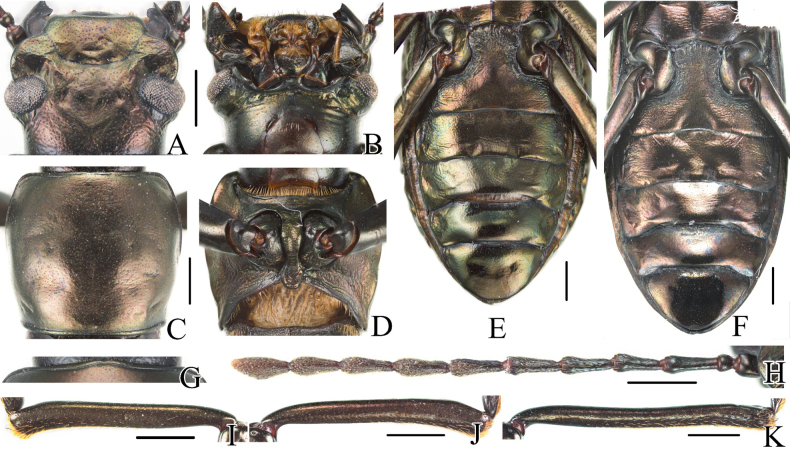
External characters of male *Morphostenophanes yangshijiani* sp. nov. Head: **A**. Dorsal view; **B**. Ventral view. Pronotum: **C**. Dorsal view; **D**. Ventral view. Male abdomens: **E**. Holotype; **F**. Paratype; **G**. Anterior margin of pronotum (male paratype); **H**. Male antenna, dorsal view; **I**. Male protibia, dorsal view; **J**. Male mesotibia, ventral view; **K**. Male metatibia, dorsal view. Scale bars: 1 mm (**A–F, H–K**); **G** not to scale.

***Head*** (Fig. 6A, B) transversely subquadrate, rugose, densely and coarsely punctate. Clypeus transversely hexagonal (width/length = 2.21), medially slightly convex, anteriorly slightly curved downward, anterior margin nearly straight, weakly emarginate in middle, clypeal transverse impression distinct to faint; frontoclypeal suture deeply grooved, becoming shallower laterally, broadly U-shaped. Genae strongly convex, rugose, strongly and roundly produced anterolaterally; outer margin deeply notched between gena and clypeus. Eyes transversely reniform, strongly convex laterally; OI = 51.4–53.2. Frons broad, smooth to rugose, anteriorly declivous towards clypeus. lateral vertexal impressions distinct. Antennae slender, reaching basal fourth of elytra; antennomeres weakly thickened to apices; relative lengths of antennomeres: 0.64: 0.29: 0.80: 0.72: 0.75: 0.78: 0.78: 0.76: 0.76: 0.72: 0.87.

***Pronotum*** (Fig. 6C, D) barrel-shaped, densely and finely punctate, PW/PL = 1.05–1.10, widest in anterior third; anterior margin nearly straight, slightly projecting in middle, anterior marginal border marked and thickened, thinning from sides toward middle and interrupted; lateral marginal borders thin, not visible in dorsal view along posterior half; posterior margin nearly straight and thickened, thinning from sides toward middle; anterior angles broadly rounded, posterior angles obtuse, disc moderately convex, with a pair of lateral impressions medially, with an elongate impression near each posterior angle. Scutellum obtusely triangular, glossy, finely punctate.

***Elytra*** elongate-oval, widest at middle, EL/EW = 1.97–2.20, strongly convex, highest near middle; with rows of striae formed by round and oval segments, areas around them with green lustre, some interrupted or confluent, encircled areas strongly convex, copper-coloured; intervals strongly convex and coppery, with fine wrinkles and fine punctures.

***Mentum*** (Fig. 6B) subquadrate; lateral margin slightly rounded; medial surface sparsely, coarsely punctate, with several large pores bearing long setae, rising gradually from sides to middle.

***Prosternum*** (Fig. 6D) green, densely rugulose, finely and sparsely punctate; prosternal process declivous, apex obtuse, dilated in ventral view; hypomeron strongly rugulose. Metasternum finely wrinkled.

***Abdomen*** (Fig. 6E) green, depressed, surface rough and weakly wrinkled, finely granulate, with sparse fine punctures; sternites III–VI wrinkled on lateral margins. Sternite III with or without a distinct posteromedian depression; sternite IV with posteromedial impressions bearing convex centres; sternite VII with a pair of shallow medial impressions.

***Protibiae*** (Fig. 6I) curved in apical sixth, thickening terminally; apical 3/5 of inner margins emarginate.

***Mesotibiae*** (Fig. 6J) as in protibiae.

***Metatibiae*** (Fig. 6K) nearly straight, weakly sinuous; apical 3/4 of inner margins sparsely pubescent.

**Figure 7.**
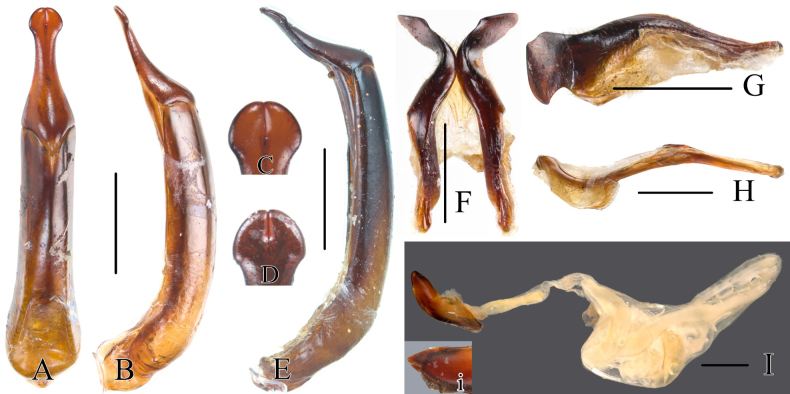
Anatomical structures of *Morphostenophanes yangshijiani* sp. nov. Aedeagus: **A**. Dorsal view, **B**. Lateral view. Enlargements of paramere apices: **C**. Holotype; **D**. Paratype; **E**. Aedeagus of paratype, lateral view. Male sternite VIII: **F**. Dorsal view; **G**. Lateral view; **H**. Spiculum gastrale, lateral view; **I**. Female reproductive organs, with close-up of ovipositor valve apices (**i**). Scale bars: 1 mm (**A–H**); 2 mm (I); **C**, **D**, **i** not to scale.

***Aedeagus*** (Fig. 7A, B) elongate, curved in lateral view; parameres slender, about 0.26 as long as total length, dorsal margin depressed medially in lateral view, apex dilated, bluntly hastate. Apical lobes of sternite VIII (Fig. 7F, G) obliquely produced posteriorly in dorsal view, subrectangular in lateral view, apical lobe with straight anterior margin, apex of superior margin slightly emarginate, inferior margins extend downward into a hook.

**Female** (Fig. 5C, E) body copper-green with a strong metallic lustre, stouter than male, length 15.5–18.4 mm, noticeably constricted between pronotum and elytra; clypeal transverse impression short, marked to absent; OI = 51.4–55.4. ***Pronotum*** with anterior margin projecting medially, disc strongly convex, and with or without a pair of shallow impressions, PW/PL = 1.10–1.22. ***Elytra*** oblong, green, broader and more convex than in male, EL/EW = 1.75–1.98, widest and highest medially, with rows of striae formed by round and oval segments, encircled areas strongly convex, areas around them with cupreous lustre, intervals strongly convex and green. ***Abdomen*** straight in lateral view, without impressions on sternites. Ovipositor shortened, abruptly narrowing terminally from apical third, with acute apex.

##### Variability.

One male paratype (Fig. 5B) differs considerably from the male holotype in both colouration and body shape; the body is aeneous and more elongate; the head and pronotum are smoother; the anterior margin of the pronotum (Fig. 6G) projects medially and the posterior margin is weakly emarginate at the middle, with a pair of elongate impressions slightly behind middle near lateral margins; and the elytra are narrowly ovate (EL/EW = 2.2) and widest and highest slightly behind the middle. Laterally, the parameres (Fig. 7E) are strongly curved ventrally, about 0.23 as long as the total length.

##### Comparative notes.

*Morphostenophanes yangshijiani* is similar to *M. minor* Zhou, 2020. Both are small species with rows of striae on the elytra formed by encircled segments. However, *M. yangshijiani* can be distinguished by its larger body size, coppery body colour (bright bronze in the latter), and encircled areas of striae strongly convex (weakly convex in the latter); the parameres are obtusely spearhead-shaped and lack an apical marginal carina (flabellate and with a carina in the latter).

##### Comments.

This species is small-bodied (<18 mm), has the protibiae curved in their apical sixth, the aedeagus is neither a broadly widened with a flabellate apex nor with an apical marginal carina, and the apical lobes of sternite VIII have hook-shaped notches on the dorsal margin in lateral view. These characters indicate that this species does not belong to the *jendeki* group. Consequently, we propose the new *yangshijiani* group for its classification.

##### Distribution.

(Fig. 4A). China: Yunnan, Jiaozishan National Nature Reserve.

##### Etymology.

The species is named after Professor Shi-jian Yang, in recognition of his assistance and support during the collection of specimens.

###### *Morphostenophanes atavus* species group (*atavus* group)

#### Morphostenophanes bannaensis

Taxon classificationAnimaliaColeopteraTenebrionidae

Zhou, 2020

FBB1D24C-B5B6-5502-9062-774AF5652776

Morphostenophanes bannaensis Zhou, 2020: 45, figs 26A–C, 31B, G, L, Q, 32B, J–L, 33B, H [male]. Type locality: Jinghong City, Mengyang Town, Yexianggu, Yunnan, China.

##### Material examined.

(females). **China**: Yunnan: • 3♀ (CHH), Jinghong City, Gasa Town, alt. 900–1500 m; vi.2023, native collector.

**Figure 8.**
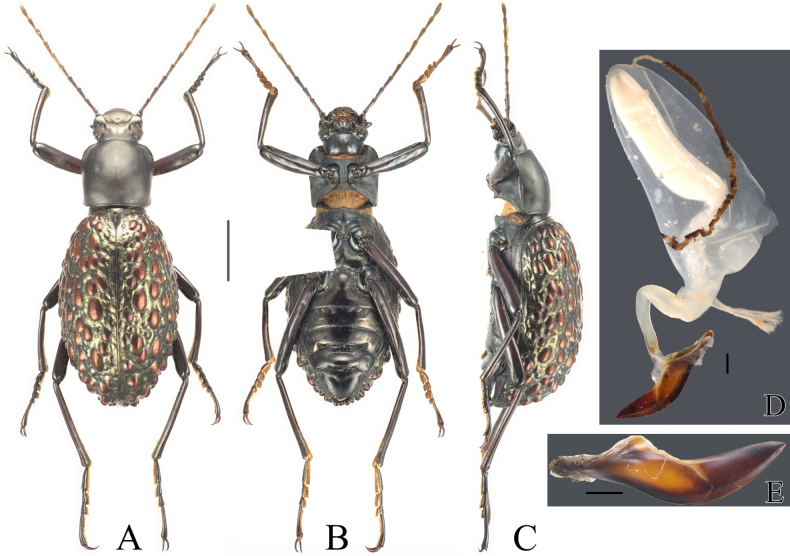
Female *Morphostenophanes bannaensis*. Habitus: **A**. Dorsal view; **B**. Ventral view; **C**. Lateral view. **D**. Female reproductive organs; **E**. Ovipositor in lateral view. Scale bars: 5 mm (**A–C**); 1 mm (**D**, **E**).

**Female** (Fig. 8A–C) body stouter than male, length 24.6–25.5 mm. width 9.60–9.74 mm; OI = 45.7–50.7; pronotum quadrate, PW/PL = 1.04–1.30; elytra stouter, EL/EW = 1.71–1.79, more convex, widest and highest medially; abdomen straight in lateral view, without impressions on sternites; ovipositor elongate, gradually narrowing apically, apex of gonocoxite acute.

**Figure 9.**
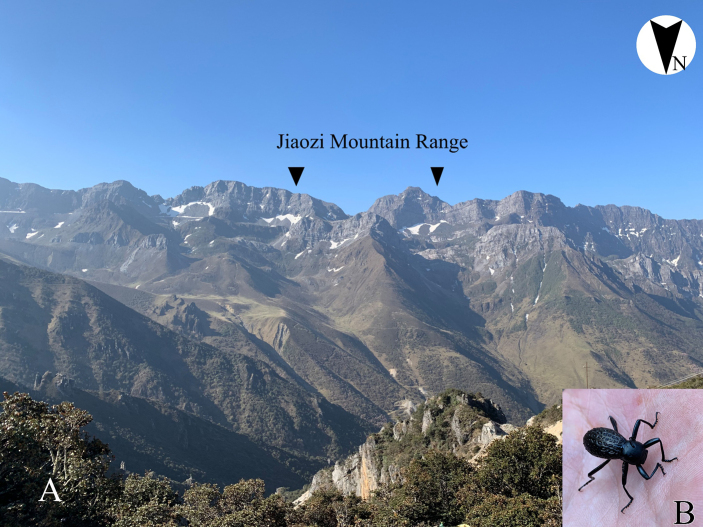
Habitat of *Morphostenophanes jiaoziensis* northern population (see Fig. 4B, yellow dot). **A**. Natural habitat in Jiaozishan National Nature Reserve, Kunming, Yunnan, alt. 3300 m; **B**. Living adult male of *M. jiaoziensis*. Photographs by Hao He, May 2022.

**Figure 10.**
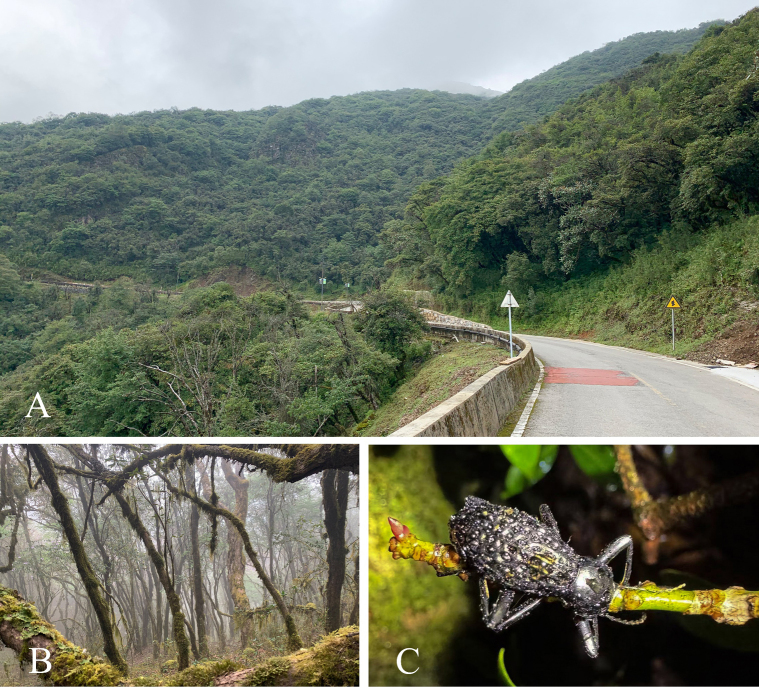
Habitat of *Morphostenophanes jiaoziensis*. **A**. Natural habitat in Jiaozishan National Nature Reserve, Kunming, Yunnan, alt. 3100 m, (see Fig. 4B, red dot); **B**. Microhabitat detail; **C**. Living adult male of *M. jiaoziensis* in situ. Photographs by Hao He: **A** July 2022; **B**, **C** June 2025.

**Figure 11.**
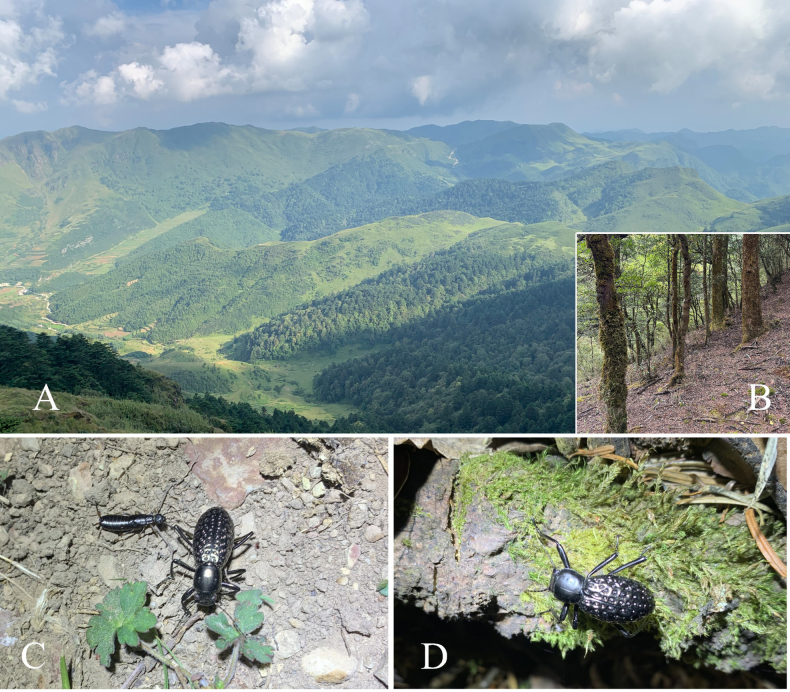
Habitat of *Morphostenophanes jiaoziensis* and *M. yangshijiani*. **A**. Natural habitat in Jiaozishan National Nature Reserve, Kunming, Yunnan, alt. 2900 m (see Fig. 4B, blue dot); **B**. Microhabitat detail; **C**. Living adult female of *M. yangshijiani* in situ; **D**. Living adult female of *M. jiaoziensis* in situ. Photographs by Hao He: July 2022 (**A**); June 2023 (**B–D**).

###### Key to the seven species groups of *Morphostenophanes*

**Table:** 

1	Elytra with continuous, straight striae, with intervals evenly convex, or elytral intervals intermittently expanded and convex, with distorted or sometimes interrupted striae	**2**
–	Elytra with interrupted striae, adjacent segments of striae usually oppositely curved, connected, and forming encircled areas with convex central portions, or with irregularly scattered, short rows of punctures, or with densely scattered, irregularly distributed tubercles	**3**
2	Body large, length > 19 mm; apical lobes of male sternite VIII weakly curved in dorsal view and without hook-shaped notches in lateral view; ovipositor elongate, gradually narrowing posteriorly	**(*atavus* group)**
–	Body small to medium-sized, length < 19 mm; apical lobes of male sternite VIII strongly curved in dorsal view, each with a hook-shaped notch in lateral view; ovipositor shortened, abruptly narrowed in apical third	**(*metallicus* group)**
3	Body small, length < 18 mm	**4**
–	Body medium-sized to large, length > 19 mm	**5**
4	Legs short; apex of parameres flabellate, apical margin evenly and abruptly thickened, forming a carina	**(*jendeki* group)**
–	Legs elongate; apical margin not thickened and never forming a carina	(***yangshijiani* group**) **7**
5	Frons rarely with medial depression, rarely depressed near eyes; elytra widest at or slightly before middle; male sternites V and VI rarely depressed in middle of apical half; ovipositor elongate, gradually narrowing posteriorly	(***elegantulus* group**) **8**
–	Frons usually depressed along midline, vaguely or markedly depressed near eyes; elytra widest at or behind middle; male sternites V and VI usually depressed in middle of apical half, with central portions of these impressions convex; ovipositor usually short, abruptly narrowed in apical third	**6**
6	Elytra with short strial irregularly scattered and interconnected punctures forming network, and intervals uneven in size and shape, some strongly swollen, forming tubercles	**(*chongli* group)**
–	Elytra with rows of annular foveae, each fovea rounded to ovate, with a convex central portion; the foveae become irregular towards the sides	**(*aenescens* group)**
**Key to species of the *yangshijiani* and *elegantulus* groups**		
7	Only one species, with the following characteristics: protibiae curved in apical sixth; apical lobes of male sternite VIII with hook-shaped notches in lateral view; ovipositor shortened, abruptly narrowed in apical third	***M. yangshijiani* sp. nov**.
8	Body dark-coloured: black or brownish black	**9**
–	Body light-coloured: bronze, greenish, or dark green	**12**
9	Body brownish black; pronotum, most of elytra, and most of legs reddish	***M. elegantulus* Masumoto & Bečvář, 2008**
–	Body uniformly black, pronotum, most of elytra, and all legs black	**10**
10	Body feebly shiny; pronotal disc without lateral depressions; elytra moderately convex in lateral view	***M. furvus* Zhou, 2020**
–	Body moderately shiny; pronotal disc with a pair of lateral depressions; elytra strongly convex in lateral view	***M. jiaoziensis* sp. nov**.
11	Width–length ratio of clypeus about 1.75; male pronotum narrower, PW/PL = 1.12–1.21; female elytra narrower, EL/EW = 1.73–1.84	***M. furvus furvus* Zhou, 2020**
–	Width–length ratio of clypeus about 2.00; male pronotum wider, PW/PL = 1.21–1.23; female elytra wider, EL/EW = 1.60–1.64	***M. furvus weishanus* Zhou, 2020**
12	Body bronze-coloured, light greenish, with weaker metallic lustre; habitus with more elongate; antennomere XI > 3× as long as wide; apex of parameres slightly bent ventrally in lateral view	***M. sinicus* Zhou, 2020**
–	Body bronze-coloured or dark green, with stronger metallic lustre; habitus less elongate; antennomere XI < 3× as long as wide; parameres straightly produced in lateral view	***M. gaoligongensis* Zhou, 2020**

## Supplementary Material

XML Treatment for Morphostenophanes jiaoziensis

XML Treatment for Morphostenophanes yangshijiani

XML Treatment for Morphostenophanes bannaensis

## Additional information

### Artificial Intelligence (AI) use

Regarding the use of AI in the preparation of this manuscript, the authors declare the following: During the preparation of this manuscript, AI-assisted tools (DeepSeek) were employed solely for language polishing purposes. AI was not involved in data analysis, result interpretation, or scientific content generation. The authors assume full responsibility for all academic content presented.

### Author ORCIDs

H. He https://orcid.org/0000-0002-8871-9671

S-J. Yang https://orcid.org/0009-0007-7917-8563

F-Y. Meng https://orcid.org/0000-0002-4522-2542

### Data availability

All of the data that support the findings of this study are available in the main text.
